# Does Size Matter to Models? Exploring the Effect of Herd Size on Outputs of a Herd-Level Disease Spread Simulator

**DOI:** 10.3389/fvets.2018.00078

**Published:** 2018-05-04

**Authors:** Mary Van Andel, Tracey Hollings, Richard Bradhurst, Andrew Robinson, Mark Burgman, M. Carolyn Gates, Paul Bingham, Tim Carpenter

**Affiliations:** ^1^Investigation and Diagnostic Centre, Surveillance and Investigation Team (Animal Health), Operations Branch, Ministry for Primary Industries, Wallaceville, New Zealand; ^2^Centre of Excellence for Biosecurity Risk Analysis, University of Melbourne, Melbourne, VIC, Australia; ^3^Centre for Environmental Policy, Imperial College London, London, United Kingdom; ^4^Epicentre, Institute of Veterinary, Animal and Biomedical Sciences, Massey University, Palmerston North, New Zealand

**Keywords:** disease spread modeling, quantitative epidemiology, biosecurity preparedness, outbreak response, animal populations

## Abstract

Disease spread modeling is widely used by veterinary authorities to predict the impact of emergency animal disease outbreaks in livestock and to evaluate the cost-effectiveness of different management interventions. Such models require knowledge of basic disease epidemiology as well as information about the population of animals at risk. Essential demographic information includes the production system, animal numbers, and their spatial locations yet many countries with significant livestock industries do not have publically available and accurate animal population information at the farm level that can be used in these models. The impact of inaccuracies in data on model outputs and the decisions based on these outputs is seldom discussed. In this analysis, we used the Australian Animal Disease model to simulate the spread of foot-and-mouth disease seeded into high-risk herds in six different farming regions in New Zealand. We used three different susceptible animal population datasets: (1) a gold standard dataset comprising known herd sizes, (2) a dataset where herd size was simulated from a beta-pert distribution for each herd production type, and (3) a dataset where herd size was simplified to the median herd size for each herd production type. We analyzed the model outputs to compare (i) the extent of disease spread, (ii) the length of the outbreaks, and (iii) the possible impacts on decisions made for simulated outbreaks in different regions. Model outputs using the different datasets showed statistically significant differences, which could have serious implications for decision making by a competent authority. Outbreak duration, number of infected properties, and vaccine doses used during the outbreak were all significantly smaller for the gold standard dataset when compared with the median herd size dataset. Initial outbreak location and disease control strategy also significantly influenced the duration of the outbreak and number of infected premises. The study findings demonstrate the importance of having accurate national-level population datasets to ensure effective decisions are made before and during disease outbreaks, reducing the damage and cost.

## Introduction

In countries that are historically free of significant livestock diseases such as foot-and-mouth disease (FMD), the outputs of disease spread models are a useful proxy for field information on disease behavior. This information may be used by the competent authority to compare the impacts of alternative disease control policy decisions (1–8). Traditionally, the policies of FMD-free countries such as the United Kingdom, the United States, Australia, and New Zealand rely on stamping out methods to eradicate outbreaks of FMD. This involves depopulation and thorough cleaning and disinfection of detected infected premises (IPs), tracing and biocontainment of all contacts, active surveillance to detect all clusters of infection, and intensive movement restrictions to limit disease spread. Areas of ongoing research include comparing the impacts of policies that would allow animals to be vaccinated, with policies that would cull animals on all affected farms. Furthermore, if a vaccination policy is considered, the impact of vaccinating cattle only compared with vaccinating all susceptible species (2) is of interest as there are seldom sufficient human resources and vaccine doses to target every animal.

These comparisons must consider the spread of disease in different regions, the effectiveness of a variety of control options, and the economic impact of the outbreak. This includes the cost of control measures and loss of trade due to restrictions implemented by the international community. The complexity of these decisions has driven the development of ever more complex disease spread simulation models that can now incorporate detailed information on within- and between-herd spread of disease. Some models, for example, use the count of animals on each farm to estimate infectivity according to latent periods, within-herd contact rates, and incubation periods, specific to the species and numbers of each species present in each herd on each farm. Although there is still much debate over the best modeling approach (2, 9–12), a key requirement of any spatially enabled disease spread simulator is national (or district/state/county)-level data of farm locations with susceptible animal populations. The model also requires data on the contact patterns of susceptible individuals and disease-specific information for each species represented. Few countries have publicly available and accurate animal population information at the farm level, which can be used in these models; however, the impact of inaccuracies in data on model outputs and decisions is seldom discussed. This is an increasingly critical point as these modeling activities generally make use of centrally held datasets, the accuracy of which is rarely scrutinized (13, 14), while modeling becomes both progressively more complex and more highly valued by decision makers.

The objective of this study was to test the null hypothesis that uncertainty around farm-level animal population sizes is not important when interpreting the outputs of within-herd spread FMD models. While no dataset can be expected to have an exact representation of herd size at every point in time, our study is concerned with examining the performance of an FMD model which explicitly models within herd spread using a heterogeneous herd dataset with census-based herd sizes, compared with simplified herd datasets where herd size is estimated according to herd type. Three different herd datasets are used in simulations that cover six geographic areas in New Zealand, under three different disease control strategies. Each of the geographic areas have large populations of foot-and-mouth susceptible livestock in different densities. Impacts on outbreak size and duration are assessed, and the potential implications for decision makers and competent authorities of using inaccurate data discussed.

## Materials and Methods

To design an experiment that provides information on the impact of herd-level animal counts on disease modeling, a disease spread simulator that utilizes the susceptible population within a herd or farm was required. The Australian Animal Disease (AADIS) model (15), which was developed in 2015 for use by the Australian Federal Government for disease response preparedness, was used for this study. AADIS is a hybrid model of livestock disease spread and control which is designed to support emergency animal disease planning. The disease simulator uses both population-based and individual-based modeling techniques. AADIS uses the herd populations to model within-herd spread with a deterministic model, and between-herd spread with a spatially explicit stochastic agent-based model (ABM). Our models use passive first IP detection which comprises two stochastic processes: detection and reporting. Detection is defined as inspecting stock (on a farm, at a saleyard, or in an abattoir), noticing clinical signs and consulting a veterinarian. An infected herd is only a candidate for detection if it meets the minimum clinical prevalence level configured for the herd type. Reporting is defined as a veterinarian suspecting FMD, sending samples to a lab, FMD being confirmed and the Chief Veterinary Officer being notified. The detection and reporting probabilities are defined per herd type and per premises type.

The AADIS model allows for representation of a situation where any type of herd may be present on any type of farm. AADIS allows disease to spread more effectively between herds on the same farm than between herds on separate farms. The ability to parameterize the spread of disease with respect to herd type and region captures the heterogeneous nature of seasonal management practices and contact patterns. For example, a beef herd on a non-commercial farm (referred to as a lifestyle block in New Zealand) will be subject to different management and marketing practices affecting the spread of disease when compared with a beef herd on a pastoral farm. AADIS also takes species and herd size into account when estimating herd susceptibility and infectivity reflecting Tildesley et al. (15) observation that a non-linear relationship between herd size and herd infectivity/susceptibility best described data from the 2001 UK FMD outbreak (15).

### Model Parameters

Herd location and herd size data were obtained from AgriBase, a commercial database of farm properties and animal populations maintained by AsureQuality, a New Zealand state owned entity (16). All AgriBase farms with no animals susceptible to FMD were removed, leaving 115,618 herds on 76,487 farms in the model (Table 1). Farms were categorized into four primary types (pastoral farming, dairy cattle farming, lifestyle farming, or pig farming) based on what farmers reported in AgriBase as their main production activity. Ten secondary herd types were created based on other livestock species present on the farm: a large and small herd type for each of deer, sheep, pigs, dairy cattle, and beef cattle, which are the farmed species in NZ susceptible to FMD (Table 1). Large or small herd type was allocated based on the farm type in AgriBase (which is specified by the farmer) to make allowance for management practices and then further divided based on the size of the herd. This made it possible for some of the effects of hobby or “lifestyle” farming to be represented by assigning a “small” herd type to those herds of any kind (or size) present on hobby farms and then allocating cut points as shown in Table 2. Cut points were chosen based on experience with farming practices in New Zealand.

**Table 1:** Counts of secondary herd types and primary farm types used to parameterize the Australian Animal Disease model.

		Primary farm type				
		Pastoral	Dairy	Pigs	Lifestyle	Total
Secondary herd types on farm	1. Large sheep	17,950				17,950
	2. Small sheep	6,772	1,526	43	12,086	20,427
	3. Large pigs	114	33	126		273
	4. Small pigs	2,044	624		1,770	4,438
	5. Large deer	3,236	63			3,299
	6. Small deer	302	22		294	618
	7. Large dairy	2,010	11,806	2		13,818
	8. Small dairy	465	24		681	1,170
	9. Large beef	23,559	1,203	40		24,802
	10. Small beef	7,907	2,080	22	18,814	28,823

	Total	64,359	17,381	233	33,645	115,618

*Movement patterns and management activities vary by herd type and susceptibility to FMD varies by species*.

**Table 2:** Descriptive data for each of the herd types used to parameterize Australian Animal Disease.

Herd type	Minimum	25th percentile	Mode	Median	Mean	75th percentile	Maximum
Large sheep	50	214.2	100	1,998.9	1,080	2,720	115,600
Small sheep	1	6	10	33.4	12	25	14,450
Large pigs	12	60	40	1,168.3	300	1,471	44,000
Small pigs	1	2	5	4.5	5	5	30
Large deer	15	76	100	404.7	187	430	19,249
Small deer	1	2	1	14.5	6	12	1,365
Large dairy	15	193	200	400.9	304	497.8	10,220
Small dairy	1	2	1	10.5	4	10	650
Large beef	15	31	20	168.1	73	195	14,500
Small beef	1	3	2	7.9	6	10	657

*The minimum, mode, and maximum were used to create the beta-pert dataset, the median for the median dataset and remaining descriptors serve to describe the distribution of the gold standard data*.

To explore the importance of herd size, three different herd size model parameterizations were derived from the AgriBase data. The first used the actual herd sizes reported in AgriBase and represented the real or “gold standard” dataset; the second assigned each herd a size equal to the median herd size for each of the 10 herd types. The third assigned each herd a size that was sampled from a beta-pert distribution generated from each of the 10 herd types. The herd type descriptive summaries used to generate the median and beta-pert distributions are shown in Table 2. Beta-pert distributions were selected to represent the herd size distributions based on testing of the gold standard herd dataset in a quantitative risk analysis software which identified this as being the best fit for the data (17). The minimum, maximum and mode (most likely) values were then selected to describe the best fitting beta-pert distribution for each of the ten herd types. Each simulation run sampled a different value for each herd from the constructed beta-pert distributions.

The disease-specific parameterization of the AADIS model was derived from the New Zealand Standard Model (NZSM) of FMD spread, which is represented in Interspread Plus, and models an outbreak of FMD serotype O pan PanAsia (18–20).

### Outbreak Seeding

Using a random seed design across the whole of New Zealand in the AADIS model results in a large degree of heterogeneity in outbreak size with an insufficient number of large outbreaks to allow comparison of the different herd size scenarios equally. Furthermore, the population densities of farms and susceptible animals are known to influence the spread of highly infectious diseases (21) as well as the efficacy of vaccination strategies for FMD (22, 23). To address these known effects, as well as to make the simulations more representative of an economically severe outbreak in New Zealand, the territorial local authorities (TLAs) that were most likely to have FMD introduced were identified and then further ranked based on where an introduced outbreak would be most likely to spread.

The greatest risks of FMD introduction to New Zealand have been reviewed and published elsewhere (24). Based on the FAO FMD contingency plans manual (25), the greatest risk for New Zealand appears to be through the feeding of FMD-infected material to non-commercially kept pigs. As there were no data available for imported materials, we used the density of small pig herds as a proxy for the risk of introduction. The likelihood of spread was based on cattle and pig population density (Table 3).

**Table 3:** Description of the six New Zealand territorial local authorities (TLAs) used as disease index herd for hypothetical foot-and-mouth (FMD) outbreaks in the study.

TLA	Area (km^2^)	Farms with FMD susceptible animals/km^2^	Mean nearest neighbor distance (km)	Count of small pig herds	Small pig herds/km^2^	Cattle/km^2^	Pigs/km^2^
New Plymouth	2,205	0.995	0.347	123	0.056	87	3.692
Auckland	4,947	1.649	0.284	240	0.049	60	2.176
Whakatane	4,474	0.27	0.454	161	0.036	38	1.504
Rangitikei	4,484	0.264	0.700	110	0.025	41	0.727
Tasman	9,650	0.201	0.534	148	0.015	13	0.049
Southland	30,198	0.11	0.862	150	0.005	22	0.038

**Figure 1:**
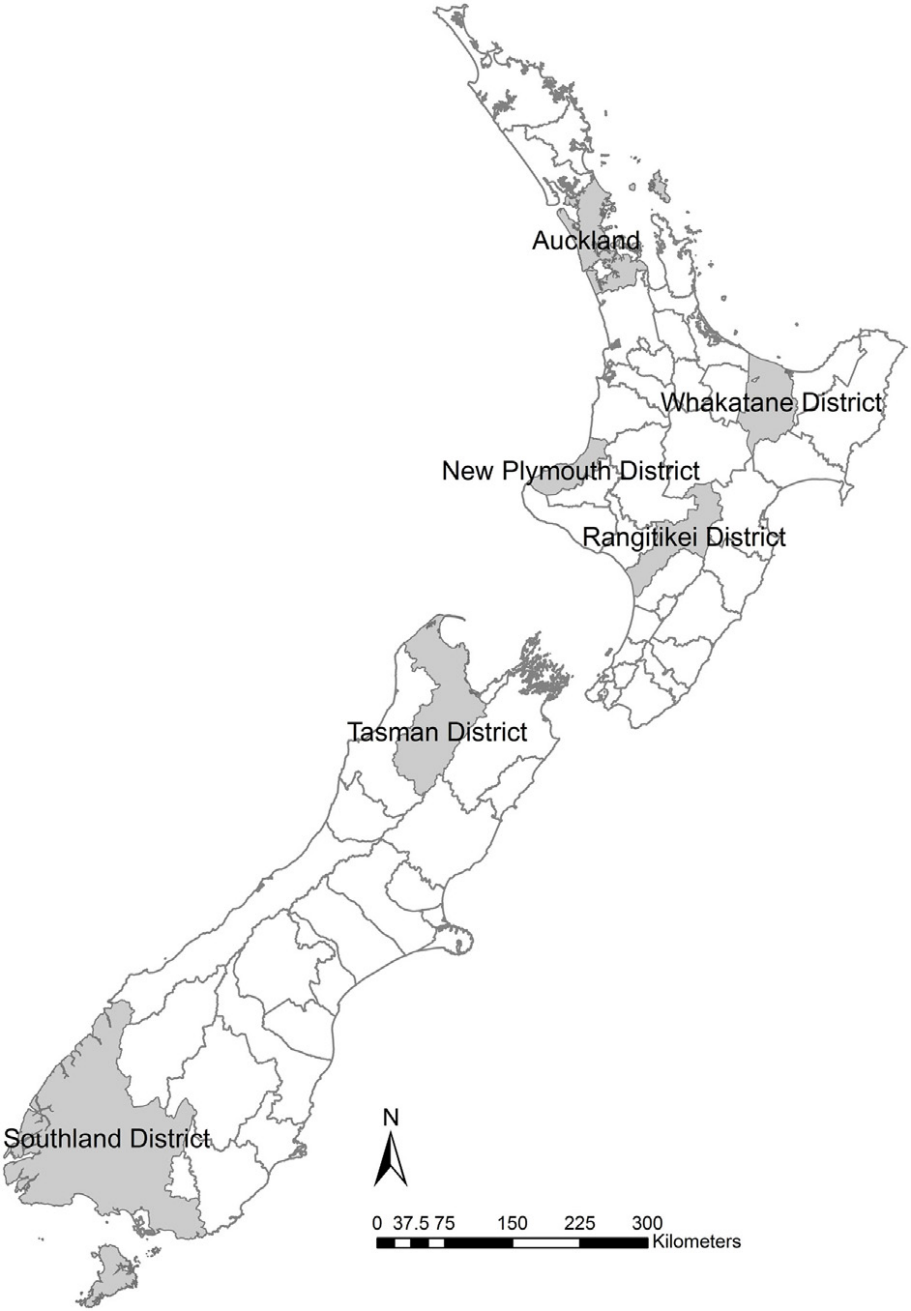
Six New Zealand territorial local authorities selected for locations of simulation models to examine the effects of variation in herd size in the within-herd spread disease model of foot-and-mouth disease, Australian Animal Disease model.

Six TLAs were chosen to provide sufficient areas to give examples in both the North and South Islands but to limit the number of TLAs so that results are still intuitively comparable (Figure 1). The goal of the study is not to predict the distribution of outbreaks sizes but to allow the comparison of the different data quality scenarios.

### Control Strategies

The control of disease within AADIS is a part of the ABM. Measures include movement restrictions, surveillance and tracing, IP operations, resource management, and vaccination. The emergent behavior of the ABM is the spatiotemporal spread of disease across the population and the subsequent activities to control and eradicate the disease. The disease spread pathways and control measures can be thought of as components of the ABM environment. Each component of the AADIS ABM environment operates independently (26). Three control strategies were modeled. The first was “stamping out” of identified infected farms (each FMD-susceptible herd on each of these farms is culled). The second applied vaccination to all susceptible species (with no restrictions on the number of doses). The third applied vaccination to cattle only.

Research is ongoing to identify alternative methods to controlling and eradicating the FMD virus, rather than automatic culling of sometimes healthy animals. The benefits of augmenting stamping out with vaccination for disease-free countries have been explored, and the strategy of vaccinating cattle only postulated as an effective alternative to vaccinating all susceptible species (2, 4, 27–30). Here we examine the effect of the accuracy of herd-level population information on the selection between two vaccination strategies, namely vaccinate cattle only, and vaccinate all susceptible animals (note that culling of animals on IPs is still employed in these strategies). When considering the use of vaccination (vs. stamping out only), decision makers must take into account the current World Organization for Animal Health (OIE) regulations, which restrict international trade for a country for an additional time period if it employs vaccination compared with if it employs a stamp out strategy (31).

### Model Simulations

One thousand simulations were performed for each of the nine model parameterizations (three control strategies and three herd populations) in each of the six selected TLAs, giving 54,000 simulations in total. The study structure is represented in Table 4. Each outbreak simulation was seeded into a small pig herd (small pig herds were identified as the most likely entry point in NZ). The same seed lists were reused in each of the 54 different model parameterizations. Simulated outbreaks, which reached 365 days’ duration (number of simulation days) were terminated.

**Table 4:** Tabular representation of a study designed to test the null hypothesis that uncertainty around herd size is not important when interpreting the results of a within-herd spread FMD model.

		Control strategy		
		Stamping out	Vaccinate all species	Vaccinate cattle only
Herd data set	Beta-pert	1A 1000 iterations in 6 TLAs	1B 1000 iterations in 6 TLAs	1C 1000 iterations in 6 TLAs
	Real	2A 1000 iterations in 6 TLAs	2B 1000 iterations in 6 TLAs	2C 1000 iterations in 6 TLAs
	Median	3A 1000 iterations in 6 TLAs	3B 1000 iterations in 6 TLAs	3C 1000 iterations in 6 TLAs

*Three control strategies and three herd population datasets were used to create nine model scenarios each of which were run for 1000 iterations in each of six New Zealand territorial local authorities—each cell (1A−3C) represents 6000 model iterations each using the same order of seed farms*.

### Statistical Analysis

Those outbreaks that failed to propagate were analyzed with a logistic regression model that included data type, TLA, and control strategy as explanatory variables and failure to propagate as the outcome variable. This analysis was performed to test the hypothesis that failure to propagate was independent of control type but was associated with herd data type and TLA.

For outbreaks that were eliminated within 365 days, outbreak duration and count of IPs on the last day of the outbreak were used as outcome variables as these are both important to decision makers choosing between control strategies. Further analysis was performed between the two vaccination scenarios using count of vaccinated animals (a proxy for vaccine doses) as the outcome variable with the same explanatory variables. We included interactions between each of these terms and a three-way interaction between all explanatory variables in all models.

Australian Animal Disease model outputs were described and analyzed using the R statistical computing language, Cox proportional hazard (CPH) models were fitted using the R survival package (32–35). The data were right censored because not all outbreaks had been eradicated within 365 days when the simulations were terminated. Although the study design is balanced, the data are not, because many simulations generated outbreaks that were not detected, and were removed from further analysis (Table 5). Therefore, CPH models were run with all orderings of predictor variables to ensure that the explanatory ability of the variables was assessed conditionally on other variables in the model.

**Table 5:** Number of simulations that generate outbreaks that are not detected (not detected), outbreaks that last 365 days without being eradicated (right censored), and number of simulations where the outbreak is detected and eradicated within 365 days (detected and eradicated), by region and by dataset.

	Beta-pert			Gold standard			Median		
	Not detected	Right censored	Detected and eradicated	Not detected	Right censored	Detected and eradicated	Not detected	Right censored	Detected and eradicated
Auckland District	1,226	4	1,770	1,066	322	1,612	1,140	1,037	823
New Plymouth District	1,200	19	1,781	745	679	1,576	692	1,576	732
Rangitikei District	1,263	0	1,737	997	173	1,830	982	758	1,260
Southland District	1,256	0	1,744	1,012	5	1,983	925	112	1,963
Tasman District	1,530	0	1,470	1,553	17	1,430	1,442	125	1,433
Whakatane District	1,337	1	1,662	811	662	1,527	928	1,304	768

In order to simplify how we determined the relative contribution of each predictor variable to the response variable, we augmented comparison of *p*-values (which are all highly statistically significant), with a comparison of the deviance values that they index. Our reasoning is that under the null hypothesis of no term effect, the deviance follows a chi-square distribution with set degrees of freedom. Ordinarily we compare these values with the index distribution to obtain a *p*-value. Because of the large numbers of simulations and the strength of the effects, all *p*-values are very small (Table 6). Our goal was to make a statement about the relative contribution of each of the experimental variables upon the response variable, but the uniformly very small *p*-values are difficult to interpret in this light. Therefore, we augmented our consideration of the predictor variables by interpreting the size of the deviance values relative to their expectation under the null hypothesis of no effect, which is the same as the number of degrees of freedom. So, the relative importance of model covariates was determined by averaging the deviance values from the output then dividing by the degrees of freedom for each term to estimate the relative deviance.

**Table 6:** Analysis of variance (ANOVA) table for the cox proportional hazards (CPH) models with infected premises (IPs) and duration as outcome variables.

Explanatory variable	Outcome variable: count of infected premises				Outcome variable: duration			
	Deviance	Chi^2^ degrees of freedom	*p*–Value	Relative deviance	Deviance	Chi^2^ degrees of freedom	*p*–Value	Relative deviance
Control type	656.4	2	<0.0001	328	519.2	2	<0.0001	260
Data type	9,937.4	2	<0.0001	4,969	10,389.4	2	<0.0001	5,195
Territorial local authority (TLA)	5,997.6	5	<0.0001	1,200	5,353.5	5	<0.0001	1,071
Control type × data type interaction	574.6	4	<0.0001	144	596.0	4	<0.0001	149
Control type × TLA interaction	478.1	10	<0.0001	48	447.8	10	<0.0001	45
Data type × TLA interaction	2,602.8	10	<0.0001	260	2,526.5	10	<0.0001	253
Control type × data type × TLA interaction	448.5	20	<0.0001	22	400.8	20	<0.0001	20

*Explanatory variables were identical for both models. Given the large size of the dataset analyzed, the small p-values might be expected, however, the large relative deviance for data type provides an indication of the importance of this variable in the models*.

To assess the effect of herd type on choice of control strategy, the strategy that had the lowest operational cost for each seed herd was recorded. This process was repeated for each of the herd data types. This allowed the percentage agreement and Fleiss and Cohen’s Kappa statistics on the lowest cost operational option to be calculated between the real, median, and beta-pert data sets (36, 37). When one of the herd type data sets did not have a completed simulation for that seed, the seed was deleted from the dataset used for comparison. This left 4,292 of a possible 6,000 data lines to compare between scenarios.

The operational cost of each strategy selected using the real herd data was compared with the operational cost that would have been incurred had an alternate strategy (based on the suggestion of the alternate herd data set) been followed. The cost of the alternate strategy was based on the cost generated by the real herd dataset. These amounts were examined as ratios rather than absolute amounts as we wish to demonstrate the value of this information rather than to predict outbreak costs which will change over time. As an example of how this comparison was made, consider a model iteration where the lowest fixed cost management strategy was to stamp out according to the real herd size dataset. This model iteration would be found in cell 2A in Table 4. Conversely, when using the median herd size data-set the lowest operational cost corresponds with the strategy to vaccinate all species in cell 3B in Table 4. To calculate the ratio between costs using the real dataset and median dataset, the real cost of the strategy to vaccinate all species (cell 2B) was divided by the cost of stamping out (cell 2 A).

## Results

Descriptive statistics for the number of simulated outbreaks that reached 365 days, the number of outbreaks that ended before they were detected, and the number of outbreaks that were detected and controlled are shown in Table 5 for each of the herd size scenarios. When the subset of simulations that ended prior to spreading were analyzed in a logistic regression model, the explanatory variables representing data type and TLA were significant, but control strategy was not significant (*p* > 0.05).

**Figure 2:**
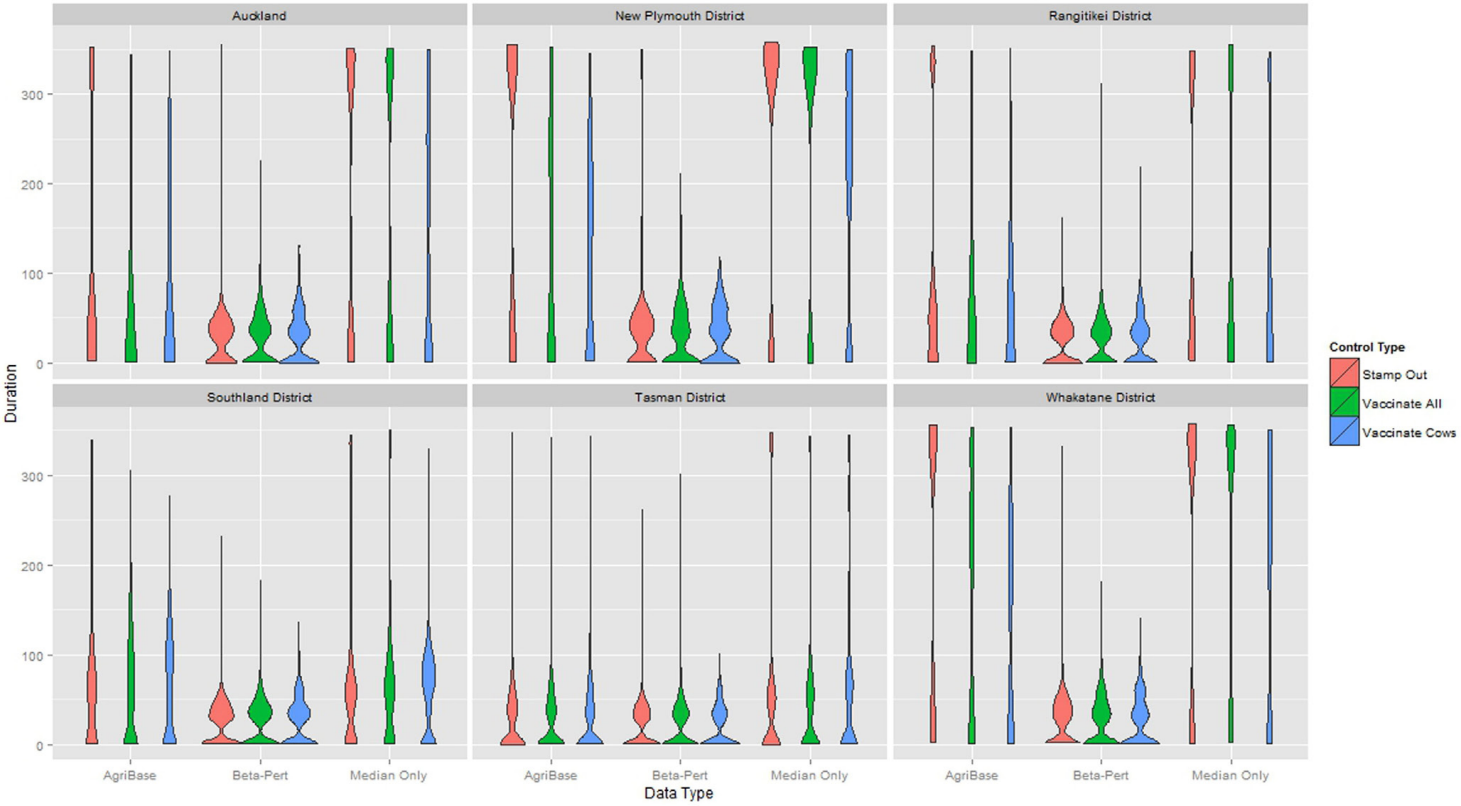
Distribution of duration of outbreak for models using each of three herd size data sets across six New Zealand territorial local authorities for three control strategies.

**Figure 3:**
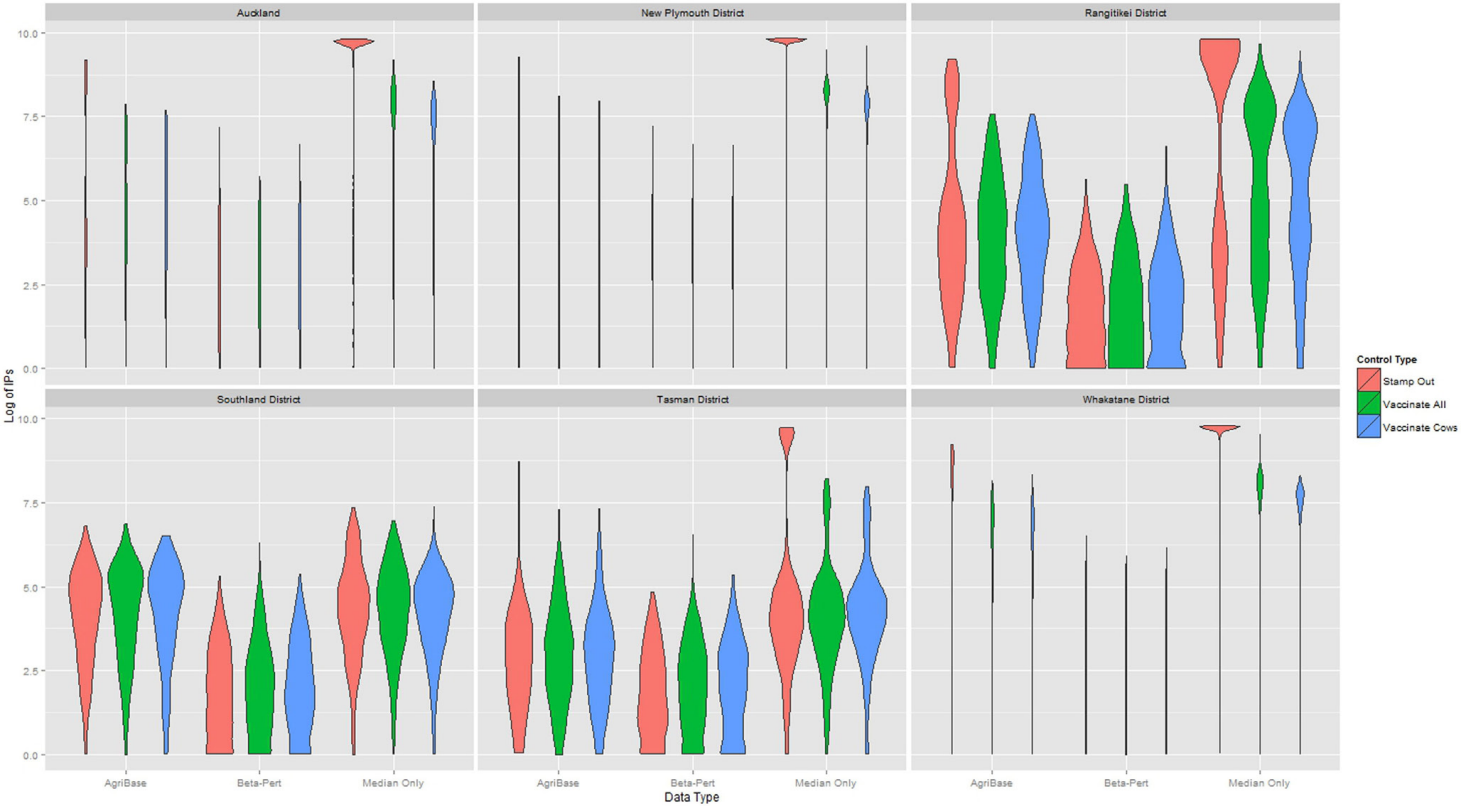
Distribution of the log10 of final count of infected premises (IPs) for models using each of three herd size data sets across six New Zealand territorial local authorities for three control strategies.

The distribution of outbreak duration and counts of IPs between TLAs, control options (only stamping out, culling IPs and vaccinating all susceptible animals, and culling IPs and vaccinating cattle only), and data sets (beta-pert modeled herd size, gold standard herd size, and median herd size) were compared and the results displayed in Figure 2 (duration) and Figure 3 (count of IPs). The CPH models demonstrated that for outbreak duration and number of IPs, all three explanatory variables are significantly associated with the outcomes. In addition, for each of the models a three-way interaction term was statistically significant, indicating that for each region both the control strategy and the data type are significantly associated with the outcome variable. This is evidenced by the relative deviance values shown in Table 6.

**Figure 4:**
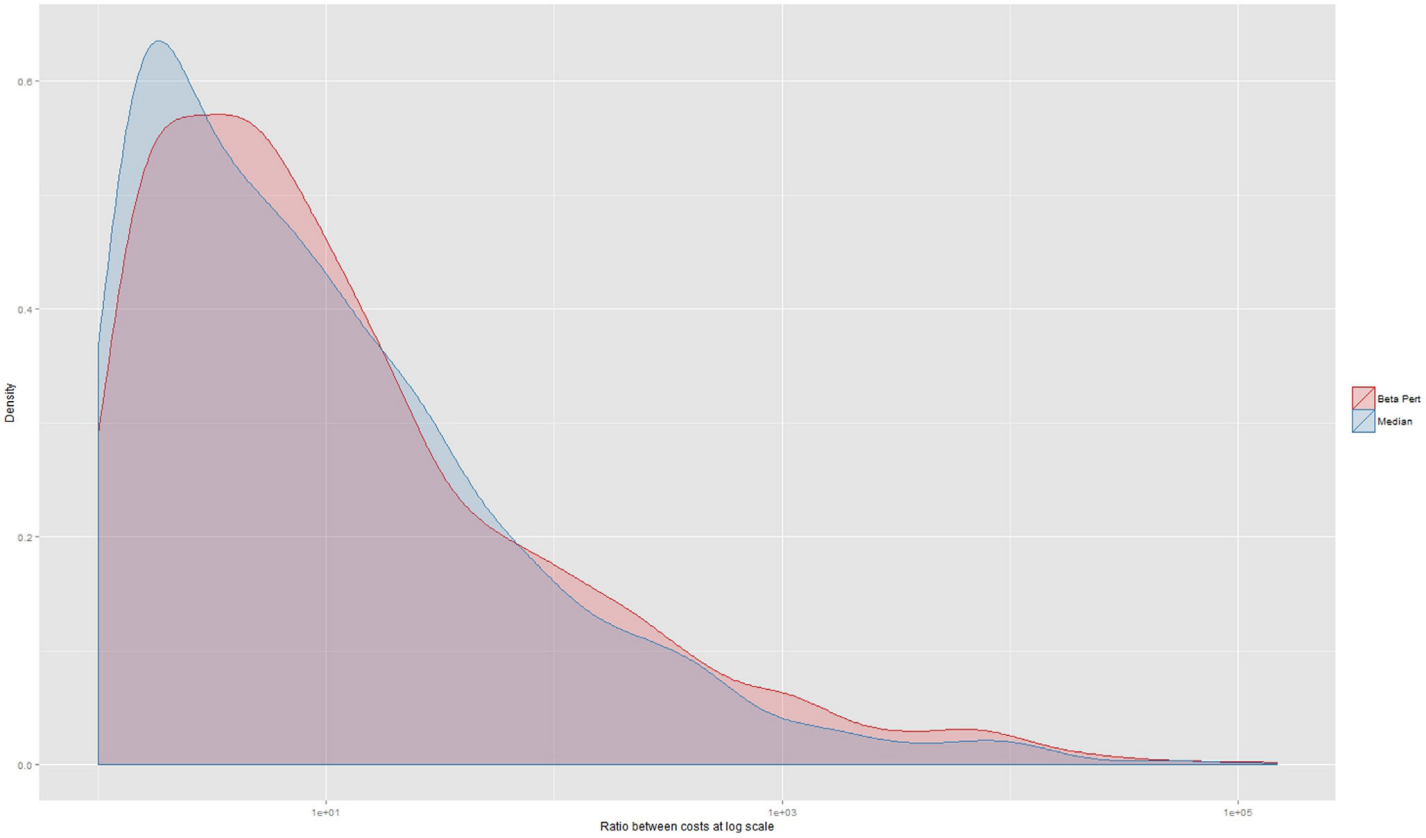
Ratio between the operational costs generated by a comparison between the strategy indicated as being the lowest by real herd data-set and the costs of the strategy that would have been chosen should an alternate herd data set have been used. The *x*-axis is displayed at the log scale. The median ratio is 7 (not at log scale) and the median of the cost ratio for those decisions using the beta-pert data-set is 7.5 and the median ratio for the decisions made using the median data-set is 6.64.

When assessing agreement between the three herd datasets on lowest operational cost strategy, 493 instances (12% of a total of 4,292 observations where each of the three herd data sets could be compared) produced the same recommendation. In 1,681 instances (39%), the simulations based on the beta-pert dataset identified the same lowest operational cost strategy as simulations based on the real dataset. Similarly, in 1,801 instances (42%), the median and real datasets identified the same strategy as being the most cost effective. A Fleiss Kappa statistic was 0.04 (*p* < 0.0001) indicating very low levels of agreement. Agreement on the most cost effective control strategy between the median and real dataset and the real and beta-pert datasets had very low Kappa statistics of 0.06 (*p* < 0.005) and 0.02 (*p* < 0.005), respectively. The size of the median ratio between the lowest cost operational strategy and the strategy indicated by the alternate (median or beta-pert) dataset was 7 (5th percentile 1.2; 25th percentile 2.5; 75th percentile 30.5; and 95th percentile 613). This distribution is shown in Figure 4 at the log scale and stratified by data type.

## Discussion

Simulation studies have been performed in the UK to identify the effect of modeled farm location information on the performance of disease spread models (38, 39). In these studies, simulations using modeled farm locations do not significantly differ from those for which farm locations are drawn from real data. To our knowledge, there are no published studies in which model results using simulated animal counts are compared with results of models using real animal counts.

The accuracy of herd population data used in modeling can significantly influence the preparations for responding to disease outbreaks. In our study, those model runs that use the gold standard herd sizes result in significantly different numbers of IPs and outbreak length when compared with those runs that used the modeled or median herd sizes. The size of the operational costs incurred based on decisions made using the median and beta-pert herd sizes was seven times the cost of the decision made using the real herd data. This result suggests that accurate population datasets should be a priority to ensure effective decisions on the best available information in order to minimize the impact of disease outbreaks. The lowest operational cost was variable among the three datasets for the same seed farm (low values of Fleiss and Cohens Kappa). Although the seed herds are the same for each instance, AADIS introduces stochasticity into each run—so true agreement may be greater than what our calculations show. Another possible source of bias is that large outbreaks that run more than 365 days without finishing were removed from the comparative data analysis.

The number of model simulations that ran for unexpectedly long durations (resulting in right censoring) is strongly correlated with data type. Those simulations run with the median herd size are over represented in all TLAs, but those TLAs that have higher density of farms with susceptible species are most affected. Similarly, a beta-pert estimation of herd size results in an over representation of outbreaks that burn out before spreading further and remain undetected (Table 5). It is possible that as the model parameterization of AADIS for New Zealand has not been fully tested and had as much time invested in it as the NZSM, it could cause artifacts in these results (18). However, as the objective of this study was to examine the impact of the accuracy of herd-level populations on a disease spread model, we focus on these findings, comparing results between models to gather information on the importance of regionally representative herd size information, rather than making recommendations on specific disease control policy for FMD free countries. Particularly important in the context of our hypothesis is the large relative deviance for data types identified in the CPH models. The relative deviance for data types is approximately five times larger than the relative deviance for the next most influential variable in the model (TLA) when considering both duration and final count of IPs.

An underestimation of outbreak size or duration in a particular region based on incorrect or estimated herd and farm population information could be as damaging to response decision making efforts as an over estimation. Take the example of the duration in days in the Whakatane region (Figure 2; Table A1 in Appendix). Here, the beta-pert data would suggest that there is little benefit in augmenting stamping out with vaccination (median of 26 days duration regardless of strategy). The median herd size data suggest that stamping out will result in longer outbreaks when using the stamping out and stamping out augmented with vaccinating all species (a median of about 330 days) when compared with stamping out augmented with vaccinating cattle only (median of 215 days). Running the model with the gold standard dataset (a more accurate reflection of regional population heterogeneity), results in a more complex picture where stamping out results in longer median outbreaks (332 days) compared with either of the vaccination augmented strategies (152 days when vaccinating all species and 138 days when vaccinating cattle only).

Actual farm population information may not be available for a variety of reasons which include resource limitation and legal restrictions (14). Where actual census data are not available, then a compromise may be to use modeled population data that are conditional on region as well as herd type rather than as a single function of herd type across the country, as has been done in this study. The optimal size for the regions that would best strike a balance between representing regional heterogeneity and best use of resource is not known. It might be argued that collection of representative samples from each locality to generate the conditional populations might require so much effort that the collection and use of actual data (which have other essential uses apart from disease spread modeling) would be a better use of resources. Our models indicate that population detail is more important in some areas of New Zealand than others and this is supported by previous work on farm animal populations in New Zealand (40). The current study does not address the impact of subtle animal count biases on disease spread as it compares only the “gold standard,” beta-pert distributed and median herd sizes for each of 10 herd types. The beta-pert representation of herd size was used based on the finding that the beta-pert distribution best fit the herd size distributions in the gold standard data. The herd type median was used as the final data set to include as InterSpread Plus, the current disease spread simulator used as the NZSM for FMD spread uses a probability of disease transmission based on the median herd size to drive disease spread in the model (18). While we have reasons for choosing both comparative datasets in our study, it is likely that inaccuracy in herd size data will, in practice not be distributed uniformly among all herds in the dataset and that some herd types will be more affected than others. We hope that our study will serve as a starting point for future, more nuanced studies which will explore the effect of sector-specific data inaccuracies.

It is important to note that no resource constraints were applied in this set of model simulations. Our results reflect that (as expected) in the absence of constraints, it is preferable to vaccinate all susceptible species than to vaccinate cattle only. Note that in Table A1 in Appendix, there appears to be a paradoxical effect of vaccinating all species in the median dataset when compared with vaccinating cattle only. This is explained by the fact that model runs that exceeded 365 days were removed from the dataset. As shown in Table 5, there are more of these model runs in the parameterizations that use the median herd size dataset which leads to larger numbers of IPs. Further investigation of the response of the model to vaccine dose and human resource limitations will make interesting future work as will further investigation of impacts not limited to operational costs of an outbreak.

Our model findings are aligned with other published research that indicates that the value of vaccination is associated with the start location of an epidemic. Furthermore, while susceptible animal density does affect outbreak size, it does not alone predict infectiousness or infectivity of a herd (22, 41). The presence of multiple species on a farm, the size of the holding and the distance to the closest infective property were risk factors identified in the 2001 UK epidemic (42). The authors point out that the accuracy of herd-level population and location information would be relevant to two (if not three) of these risk factors. In addition, should an actual outbreak occur, the strain type of the virus and its specific epidemiology would be hugely influential on the effectiveness of any chosen control strategy (20).

Our study indicates that when using a disease spread simulator that explicitly represents the spread of disease within farms the quality and origin of the data used to represent herd size has significant impacts on the model results. We recommend that specific attention needs to be focused on national-level animal population datasets that results in their alignment and more efficient utilization.

## Author Contributions

MA parameterized and ran the AADIS model. RB provided AADIS support and advice and made necessary code changes for AADIS to be able to dynamically define herd sizes *via* a distribution. AR, TH, and MA performed statistical analysis of the results. MG, TC, and MA designed the study. All authors contributed to writing the manuscript.

## Conflict of Interest Statement

The authors declare that the research was conducted in the absence of any commercial or financial relationships that could be construed as a potential conflict of interest.

## Appendix

**Table A1:** Descriptive five number summaries (minimum, 25th percentile, median, mean, 75th percentile and maximum) for each of 2 outcome variables (count of infected premises and duration in days) and 3 explanatory variables for the models described in Table 4.

Data type	Outcome variable	Region	Stamping out (minimum, 25th percentile, median, mean, 75th percentile, maximum)	Stamping out augmented with vaccinating all susceptible species (minimum, 25th percentile, median, mean, 75th percentile, maximum)	Stamping out augmented with vaccinating cattle only (minimum, 25th percentile, median, mean, 75th percentile, maximum)
Gold standard herd size data	Infected Premises	Auckland District	0, 0, 19, 1658, 679.5, 10320	0, 0, 18.5, 264.2, 199, 2729	0, 0, 21.5, 275.9, 334.2, 2275
		New Plymouth District	0, 1, 2702, 3823, 8149, 11190	0, 1, 284, 591.6, 1073, 3394	0, 0, 284, 554.8, 994.8, 2961
		Rangitikei District	0, 0, 14, 644.5, 103, 10630	0, 0, 12, 119.3, 93.5, 1956	0, 0, 14, 122.2, 95, 1997
		Southland District	0, 0, 23, 83.08, 118, 928	0, 0, 20, 85.73, 129, 957	0, 0, 25, 89.42, 134.2, 692
		Tasman District	0, 0, 0, 59.53, 16.25, 6454	0, 0, 0, 24.3, 17, 1462	0, 0, 0, 29.41, 17, 1580
		Whakatane District	0, 0, 2120, 3531, 7187, 10460	0, 0, 245.5, 609.4, 1175, 3553	0, 0, 254.5, 561.9, 1079, 4320
	
	Duration in days	Auckland District	0, 0, 42.5, 109.5, 321.5, 355	0, 0, 40, 83.13, 123, 346	0, 0, 47.5, 85.71, 154, 349
		New Plymouth District	0, 27, 335, 201.2, 342, 357	0, 23.75, 128.5, 140.9, 247.2, 354	0, 0, 127.5, 126.8, 209, 347
		Rangitikei District	0, 0, 47, 87.34, 90, 355	0, 0, 45, 69, 103.2, 348	0, 0, 55, 69.79, 106.2, 352
		Southland District	0, 0, 54, 63.25, 89, 340	0, 0, 52, 59.38, 98, 306	0, 0, 61, 62.54, 106, 278
		Tasman District	0, 0, 0, 29.84, 47, 347	0, 0, 0, 28.34, 45, 342	0, 0, 0, 29.64, 46, 343
		Whakatane District	0, 0, 332, 193.6, 342, 357	0, 0, 152, 147.6, 258, 355	0, 0, 137.5, 130.5, 224, 354

Beta pert modelled herd size data	Infected Premises	Auckland District	0, 0, 3, 26.62, 24, 1350	0, 0, 3, 24.3, 26, 313	0, 0, 3, 25.49, 25.25, 796
		New Plymouth District	0, 0, 5, 53.37, 50.25, 1368	0, 0, 2, 33.94, 32, 805	0, 0, 4, 37.55, 39, 785
		Rangitikei District	0, 0, 1, 7.771, 7, 283	0, 0, 1, 10.51, 8, 244	0, 0, 1, 11.19, 8, 749
		Southland District	0, 0, 1, 8.901, 9, 206	0, 0, 1, 10.66, 9, 567	0, 0, 1, 10.1, 9, 222
		Tasman District	0, 0, 0, 6.898, 6, 129	0, 0, 0, 8.579, 7, 707	0, 0, 0, 7.982, 7, 214
		Whakatane District	0, 0, 1, 18.11, 16, 691	0, 0, 1, 16.52, 16, 373	0, 0, 1, 16.19, 14, 492
	
	Duration in days	Auckland District	0, 0, 30, 27.18, 43, 356	0, 0, 30, 27.78, 44, 226	0, 0, 29.5, 27.59, 45, 131
		New Plymouth District	0, 0, 34, 37.63, 50, 351	0, 0, 29, 30.9, 53, 212	0, 0, 34, 32.04, 57, 119
		Rangitikei District	0, 0, 27, 23.32, 40, 162	0, 0, 28, 25.63, 40, 312	0, 0, 27, 25.46, 41, 219
		Southland District	0, 0, 27, 23.4, 40, 232	0, 0, 28, 23.99, 40, 183	0, 0, 28, 24.88, 40, 137
		Tasman District	0, 0, 0, 18.96, 36, 263	0, 0, 0, 20.03, 37, 301	0, 0, 0, 19.52, 38, 101
		Whakatane District	0, 0, 26, 24.82, 42, 333	0, 0, 26, 25.65, 42.25, 181	0, 0, 25, 24.95, 43, 141

Median herd size data	Infected premises	Auckland District	0, 0, 9510, 8281, 16750, 19160	0, 0, 207, 1365, 2816, 10220	0, 0, 173, 908.5, 1877, 5328
		New Plymouth District	0, 14, 17250, 12890, 17890, 19330	0, 13.5, 3108, 2660, 4128, 13800	0, 8.25, 2029, 1765, 2765, 14850
		Rangitikei District	0, 0, 33.5, 5657, 14700, 19220	0, 0, 27.5, 760.2, 1062, 16410	0, 0, 36, 465.6, 682.2, 13040
		Southland District	0, 0, 29, 114.7, 123.2, 1550	0, 0, 33, 98.43, 124.2, 1079	0, 0, 37, 77.89, 114, 1648
		Tasman District	0, 0, 4.5, 981.9, 63, 17980	0, 0, 2, 113.9, 59, 3877	0, 0, 2, 101.2, 68, 2969
		Whakatane District	0, 0, 15780, 10220, 17210, 18990	0, 0, 1902, 1843, 3334, 14150	0, 0, 1426, 1301, 2290, 4115
	
	Duration in days	Auckland District	0, 0, 326, 180.6, 341, 352	0, 0, 122, 167.5, 339, 353	0, 0, 108.5, 137.8, 261, 350
		New Plymouth District	0, 44, 340, 254.5, 343.2, 358	0, 57.25, 340, 247.3, 343, 352	0, 40, 230.5, 194.9, 294, 351
		Rangitikei District	0, 0, 58, 150, 336, 350	0, 0, 58.5, 130.4, 328, 357	0, 0, 66, 110.4, 213, 348
		Southland District	0, 0, 52, 71.41, 77.25, 346	0, 0, 57, 70.34, 86.25, 350	0, 0, 66, 56.13, 87, 330
		Tasman District	0, 0, 34, 56.34, 61, 348	0, 0, 28.5, 47.4, 63, 345	0, 0, 28.5, 46, 69, 345
		Whakatane District	0, 0, 337, 212.2, 343, 358	0, 0, 334, 206.6, 342, 358	0, 0, 215, 171.1, 284.2, 351

*These results are graphically represented in Figures 2 and 3*.
